# Liver Function Tests and Risk Prediction of Incident Type 2 Diabetes: Evaluation in Two Independent Cohorts

**DOI:** 10.1371/journal.pone.0051496

**Published:** 2012-12-17

**Authors:** Ali Abbasi, Stephan J. L. Bakker, Eva Corpeleijn, Daphne L. van der A, Ron T. Gansevoort, Rijk O. B. Gans, Linda M. Peelen, Yvonne T. van der Schouw, Ronald P. Stolk, Gerjan Navis, Annemieke M. W. Spijkerman, Joline W. J. Beulens

**Affiliations:** 1 Department of Epidemiology, University of Groningen, University Medical Center Groningen, Groningen, The Netherlands; 2 Department of Internal Medicine, University of Groningen, University Medical Center Groningen, Groningen, The Netherlands; 3 Julius Center for Health Sciences and Primary Care, University Medical Center Utrecht, Utrecht, The Netherlands; 4 Center for Nutrition and Health, National Institute for Public Health and the Environment (RIVM), Bilthoven, The Netherlands; 5 Center for Prevention and Health Services Research, National Institute for Public Health and the Environment (RIVM), Bilthoven, The Netherlands; German Diabetes Center, Leibniz Center for Diabetes Research at Heinrich Heine University Duesseldorf, Germany

## Abstract

**Background:**

Liver function tests might predict the risk of type 2 diabetes. An independent study evaluating utility of these markers compared with an existing prediction model is yet lacking.

**Methods and Findings:**

We performed a case-cohort study, including random subcohort (6.5%) from 38,379 participants with 924 incident diabetes cases (the Dutch contribution to the European Prospective Investigation Into Cancer and Nutrition, EPIC-NL, the Netherlands), and another population-based cohort study including 7,952 participants with 503 incident cases (the Prevention of Renal and Vascular End-stage Disease, PREVEND, Groningen, the Netherlands). We examined predictive value of combination of the Liver function tests (gamma-glutamyltransferase, alanine aminotransferase, aspartate aminotransferase and albumin) above validated models for 7.5-year risk of diabetes (the Cooperative Health Research in the Region of Augsburg, the KORA study). Basic model includes age, sex, BMI, smoking, hypertension and parental diabetes. Clinical models additionally include glucose and uric acid (model1) and HbA1c (model2). In both studies, addition of Liver function tests to the basic model improved the prediction (C-statistic by∼0.020; NRI by∼9.0%; P<0.001). In the EPIC-NL case-cohort study, addition to clinical model1 resulted in statistically significant improvement in the overall population (C-statistic = +0.009; P<0.001; NRI = 8.8%; P<0.001), while addition to clinical model 2 yielded marginal improvement limited to men (C-statistic = +0.007; P = 0.06; NRI = 3.3%; P = 0.04). In the PREVEND cohort study, addition to clinical model 1 resulted in significant improvement in the overall population (C-statistic change = 0.008; P = 0.003; NRI = 3.6%; P = 0.03), with largest improvement in men (C-statistic change = 0.013; P = 0.01; NRI = 5.4%; P = 0.04). In PREVEND, improvement compared to clinical model 2 could not be tested because of lack of HbA1c data.

**Conclusions:**

Liver function tests modestly improve prediction for medium-term risk of incident diabetes above basic and extended clinical prediction models, only if no HbA1c is incorporated. If data on HbA1c are available, Liver function tests have little incremental predictive value, although a small benefit may be present in men.

## Introduction

Change in liver function tests is considered as surrogate marker of liver injury and nonalcholic fatty liver disease (NAFLD) [1]. Previous studies have demonstrated that circulating concentration of liver function tests like gamma-glutamyltransferase (GGT), alanine aminotransferase (ALT) and aspartate aminotransferase (AST) are increased in individuals with insulin resistance and the metabolic syndrome [1]–[3]. In addition, these components of liver function tests have been shown to be positively associated with the risk of future type 2 diabetes [1], [4]. A recent meta-analysis on this topic showed that both elevated ALT and GGT were associated with increased risk of diabetes, while GGT might be a stronger risk factor than ALT [4].

However, there is only a limited number of studies that examine the predictive value of liver function tests for the risk of future diabetes in terms of essential measures of prediction, such as the C-statistic to assess discrimination between people who develop diabetes and those who don’t [5]–[8]. These studies mainly developed [6], [7] or updated [5] clinical prediction models by incorporating one or two components of liver function tests in each models. It is important to note that the predictive value of liver function tests was examined in combination with other (bio)markers and in the same data set that was used to develop the original models [5]–[7]. Of these, 2 studies showed improvement in prediction when GGT plus glycaemia indices were added to a basic model consisting only of data that can be derived without need for taking blood samples [6], [7]. In another study, a combination of GGT, ALT, triglycerides and HDL cholesterol improved discrimination above a diabetes risk score including HbA1c and glucose [5].

So, whether liver function tests have incremental predictive value above validated model(s) is still unclear. An independent study evaluating utility of these markers of liver function when incorporated in an existing prediction model is needed to answer this question [9], [10]. Recently, we validated and updated German prediction models from the Cooperative Health Research in the Region of Augsburg (KORA) study in a Dutch general population cohort [11], [12]. In the current study, we addressed the incremental predictive value of liver function tests for the risk of future type 2 diabetes when compared with the KORA models [12]. To do so, we analysed data from two independent cohorts separately. In each cohort, we performed analyses in the total population and sex-stratified subgroups to account for potential sex differences in the prediction performance of each model [6], [11], [13].

## Research Design and Methods

### Study Design and Populations

We used data from two cohorts of general population in the Netherlands: 1) the Dutch contribution to the European Prospective Investigation Into Cancer and Nutrition (EPIC-NL) study; and 2) the Prevention of Renal and Vascular End-stage Disease (PREVEND) study. Details of each study design and recruitment of participants have been published previously [14], [15].

In brief, the EPIC-NL cohort (n = 40,011) includes the Monitoring Project on Risk Factors for Chronic Diseases (MORGEN) and Prospect cohorts, initiated between 1993 and 1997. Prospect is a prospective cohort study of 17,357 women aged 49–70 years who participated in a breast cancer screening programme. The MORGEN cohort consists of 22,654 men and women aged 20–64 years who were recruited through random population sampling in three Dutch towns (Amsterdam, Maastricht and Doetinchem). A new random sample of about 5,000 participants was examined each year. We excluded 615 individuals with prevalent type 2 diabetes and 1,017 with missing follow-up or who did not consent to linkage with disease registries, leaving 38,379 individuals in the full cohort. In a 6.5% baseline random sample (n = 2,604) with biochemical measurements [14], similar exclusion criteria were applied. After exclusions, 2,506 individuals (including 79 incident diabetes cases) from the random sample and 924 incident diabetes cases in the full cohort remained for the case-cohort study [16]. We used this case-cohort sample for all analyses.

In brief, the baseline PREVEND cohort (n = 8,592) was recruited from inhabitants (aged 28–75 years) of the city of Groningen, the Netherlands. Baseline measurements were performed between 1997 and 1998. The PREVEND cohort included a total of 6,000 individuals with a morning urinary albumin concentration of 10 mg/l or greater and a random control sample of individuals with a urinary albumin concentration of less than 10 mg/L (n = 2,592). Overall, we excluded 336 individuals with prevalent type 2 diabetes and 277 with missing data on follow-up, leaving 7,979 individuals for the full cohort study. We used this full cohort sample for all analyses.

### Ethics Statement

All participants gave written informed consent prior to study inclusion. All cohort studies complied with the Declaration of Helsinki and were approved by local medical ethics committees.

### Measurements of Biomarkers

In the EPIC-NL study, the general questionnaire contained questions on demographic characteristics and risk factors for the presence of chronic diseases. Body weight, height and waist and hip circumference were measured according to standard procedures. Hypertension was defined based on self-report of diagnosis by a physician, measured hypertension (≥140 mmHg systolic blood pressure or ≥90 mmHg diastolic blood pressure) or the use of blood pressure-lowering medication. Non-fasting blood samples were collected at baseline from all participants. HbA1c was measured in erythrocytes using an immunoturbidimetric latex test. Glucose and uric acid were measured using enzymatic methods. AST, ALT and GGT were measured using enzymatic methods and albumin by a colorimetric method [14].

In the PREVEND study, the participants underwent two outpatient visits to assess baseline data on demographics, anthropometric measurements, cardiovascular risk factors, health behaviours, and medical family history and to collect two 24-hour urine samples on 2 consecutive days. Blood pressure values are given as the mean of the last two recordings of both visits as this provides the values after stabilization of blood pressure. Plasma glucose was measured by dry chemistry (Eastman Kodak, Rochester, New York). All liver function tests were measured by a standardized enzymatic method (Modular P; Roche Diagnostics, Indianapolis, IN).

### Definition of Main Outcome

In the EPIC-NL study, potential incident type 2 diabetes was self-reported via two follow-up questionnaires at 3- to 5-year intervals in the MORGEN and Prospect cohort. In the Prospect cohort, a urinary glucose strip test was sent along with the first follow-up questionnaire as a screening method. Diagnoses of type 2 diabetes were also obtained from the Dutch Center for Health Care Information, which holds a standardized computerized register of hospital discharge diagnoses. Follow-up was complete until January 1, 2006. Potential cases identified by these methods were verified against general practitioner (MORGEN and Prospect) or pharmacist records (Prospect only). We defined type 2 diabetes as being present when the diagnosis was confirmed by either of these methods. For 89% of participants with potential diabetes, verification information was available, and 72% were verified as having type 2 diabetes and were included as cases of type 2 diabetes in this analysis [17]. The rest of individuals were considered as non-cases.

In the PREVEND study, incident cases of diabetes were ascertained as described previously [18]. In brief, incident diabetes was considered present if one or more of the following criteria were met: 1) a fasting plasma glucose of ≥7.0 mmol/l (126 mg/dl) or random sample plasma glucose ≥11.1 mmol/l (200 mg/dl); 2) self-reported physician’s diagnosis; 3) use of glucose-lowering agents according to a central pharmacy registration.

### Statistical Analysis

First, we examined the association between the components of liver function tests (including GGT, ALT, AST and albumin) and the risk of future diabetes. For liver function tests, we used logarithm transformation with base 2 (log_2_) to allow for interpretation of results per increase of 100% of values of each component. We used Cox proportional-hazards regression in the EPIC-NL study which was adapted for case-cohort analysis. We used logistic regression in the PREVEND full cohort study, because the events have been detected at regular screening visits or shortly thereafter. Thus, estimated survival and hazards can not be accurately calculated by this type of follow-up. In step 1, we calculated age and sex-adjusted hazard ratios (HRs) and odd ratios with 95% CIs for the risk of diabetes by doubling of concentrations of each liver function tests (per log_2_ unit increase). In step 2, we adjusted for age, sex, parental diabetes, body mass index (BMI), smoking status, hypertension, glucose and uric acid. In step 3, we further adjusted for HbA1c. This could only be done in the EPIC-NL case-cohort study, because data on HbA1c were not available in the PREVEND study.

To account for the case-cohort design in the EPIC-NL study, we applied an extrapolation approach which extends the case-cohort data to the size of the entire cohort [19]. This is achieved by extrapolating the non-cases of the random sample (i.e., total random sample of 2,506 individuals minus 79 cases) to the number of non-cases in the full cohort (i.e., total sample of 38,379 individuals minus 924 cases). To do so, we substituted the non-cases of the full cohort (n = 37,455) by a random multiplication of non-cases of the random sample (n = 2,427). We have previously described and validated this approach [20].

In the second part of this study, we computed the probability of getting diabetes using the KORA basic model which was previously validated and updated in the PREVEND cohort [11]. As previously described [11], we recalibrated the original KORA model by means of logistic regression to derive the intercept and the calibration slope in the PREVEND cohort study. We also adjusted for the difference in incident diabetes between the KORA and the EPIC-NL cohorts by fitting the original KORA model in the EPIC-NL case-cohort study [21]. Figure S1 (a, b) depicts the agreement between the predicted 7.5-year risk and observed risk of type 2 diabetes after recalibration in each cohort. The basic model included data on age, sex, parental diabetes, body mass index (BMI), smoking status and hypertension [12]. Clinical model 1 included additional data on glucose plus serum uric acid; and in clinical model 2 we further added HbA1c. As the original KORA models have been developed for a time period of risk prediction of 7.5 years, we examined the incremental predictive value of liver function tests also for the 7.5-year risk of developing type 2 diabetes. Therefore, participants who developed diabetes after more than 7.5 years of follow-up were included in 7.5-year prediction as non-cases. We examined added predictive value of 1) each component alone, 2) combination of GGT+ALT and 3) a panel of GGT, ALT, AST and albumin. We assessed improvement of type 2 diabetes prediction in terms of discrimination by calculating the C-statistic with 95%CI, and reclassification by calculating integrated discrimination improvement (IDI) and net reclassification improvement (NRI) [22], [23]. To calculate the NRI, cut-off values for risk categories have to be defined. In previous studies, a number of risk categories for the 10-year risk of cardiovascular disease [23], [24] or type 2 diabetes [25], [26] have been reported. In the present study, we slightly modified these cut-off values according to the shorter time period (and hence the lower average observed risk) [25], [26], using cut-off values of <4% for low-risk, 4%–8% for intermediate-risk and ≥8% for high-risk.

In the EPIC-NL case-cohort study, for most predictors <1% of data were missing; however, missing values occurred in 5% for parental history of diabetes, and 20.5% for glucose levels. Because an analysis of only the completely observed data may often lead to biased results, we imputed these missing values using single imputation and predictive mean matching [27]. As the percentage of missing values for the non-fasting glucose concentration was relatively high, we repeated our analyses using only data from the MORGEN cohort, in which less than 10% of values for non-fasting glucose concentration were missing, as a sensitivity analysis. In the PREVEND cohort study, for most variables, <1% were missing, whereas this was up to 7.5% for self-reported variables. To account for missing values, we used a similar approach to that of the EPIC-NL study. Table S1 in supporting information shows the number of missing values for all variables incorporated in each model. We also used a weighted method to compensate for baseline enrichment of the PREVEND participants with high urinary albumin concentration (>10 mg/l). All the statistical analyses were carried out using IBM SPSS 19.0 and R version 2.13.1 (Vienna, Austria) for Windows (http://cran.r-project.org/).

## Results

### Baseline Clinical Characteristics

We summarize baseline characteristics of the participants of each study in Table 1. Participants of the EPIC-NL study were more likely to be women, and to have hypertension and parental history of diabetes, whereas participants of the PREVEND study were more likely to be smoker and had slightly higher uric acid and albumin on average. In the EPIC-NL cohort study, we ascertained and validated 924 (2.4%) incident cases of type diabetes during a median follow-up of 10.2 years (over 387,000 person-years). In the PREVEND cohort study, we ascertained 503 (6.3%) incident cases during a median follow-up of 7.7 years (over 60,186 person-years).

**Table 1:** Baseline participants’ characteristics in each study.*

	EPIC-NL study			PREVEND study	
Variables	Full cohort	Random sample	Cases with incident type 2 diabetes	Full cohort	Cases with incident type 2 diabetes
No. of individuals	38379	2506	924	7979	503
Age– yr	49.1 (11.9)	49.2 (11.9)	56.6 (7.3)	48.9 (12.5)	56.5 (10.7)
Female gender– no. (%)	28531 (74.3)	1872 (74.7)	722 (78.1)	4065 (50.9)	210 (41.7)
Parental history of diabetes– no. (%)	7379 (19.2)	498 (19.9)	377 (40.8)	1252 (15.7)	143 (28.4)
Hypertension– no. (%)†	14122 (36.8)	953 (38.0)	626 (67.7)	2248 (28.2)	275 (54.7)
Antihypertensive medication – no. (%)	3736 (9.7)	259 (10.3)	275 (29.8)	1036 (13.0)	133 (26.4)
Current smoker– no. (%)	11749 (30.6)	789 (31.5)	322 (34.8)	2742 (34.4)	174 (34.6)
Exsmoker– no. (%)	12004 (31.3)	769 (30.7)	241 (26.1)	2896 (36.3)	205 (40.8)
Body­mass index‡	25.6 (4.0)	25.7 (4.0)	29.9 (4.7)	26.0 (4.2)	29.5 (4.7)
Waist cimrcumfernce– cm	85.1 (11.4)	85.3 (11.6)	97.0 (11.6)	88.1 (12.9)	99.2 (12.2)
Systolic blood pressure– mm Hg	126.0 (18.8)	126.1 (18.6)	139.9 (21.6)	123.9 (19.3)	135.7 (20.4)
**Biomarkers**					
Glucose– mmol/liter§	4.9 (1.2)	4.89 (1.17)	6.75 (2.48)	4.7 (0.6)	5.6 (0.7)
HbA1c– %	–	5.39 (0.58)	6.5 (1.4)	–	–
Uric acid–µmol/l	–	259.42 (68.48)	285.76 (71.04)	303.07 (80.16)	355.13 (81.94)
GGT– U/liter	–	25.5 (20.0)	36.6 (28.9)	32.6 (45.6)	53.9 (126.7)
ALT– U/liter	–	20.2 (11.9)	20.3 (11.8)	23.9 (20.7)	31.3 (40.6)
AST– U/liter	–	22.7 (9.1)	22.7 (9.0)	25.7 (10.4)	28.7 (19.4)
Albumin– g/L	–	38.9 (4.9)	37.1 (4.9)	45.8 (2.7)	45.4 (3.0)

* Data were shown as mean (SD) for continuous variables, and numbers (percentage) for categorical variables. EPIC-NL denotes Dutch contribution of the European Prospective Investigation Into Cancer and Nutrition, PREVEND denotes Prevention of Renal and Vascular End-stage Disease, HbA1c glycated hemoglobin, GGT gamma-glutamyltransferase, ALT alanine aminotransferase and AST aspartate aminotransferase.

† Hypertension was ascertained on the basis of self-reported diagnosis by a physician, antihypertensive medication use, systolic blood pressure ≥140 mm Hg, or diastolic blood pressure ≥90 diastolic blood pressure, or a combination of these.

‡ Body mass index is the weight in kilograms divided by the square of the height in meters.

§ To convert values for glucose to milligrams per deciliter, divide by 0.0555. To convert values for uric acid to milligrams per deciliter, divide by 59.48.

### Liver Function Tests and Type 2 Diabetes

Table 2 depicts the associations between components of liver function tests and the risk of diabetes, calculated per 100% increase of marker concentrations in total populations and in sex-stratified subgroups. In the EPIC-NL case-cohort study, the multivariable-adjusted HRs (95%CI) for the risk of diabetes were 1.49 (1.37–1.61), 1.22 (1.09–1.37), 0.97 (0.81–1.17) and 0.34 (0.21–0.54) per doubling concentrations of GGT, ALT, AST and albumin, respectively. In the PREVEND cohort study, the multivariable-adjusted ORs (95%CI) for the risk of diabetes were 1.22 (1.09–1.38), 1.29 (1.11–1.50), 1.16 (0.89–1.50) and 0.31 (0.87–1.05) per 100% increase of concentrations of GGT, ALT, AST and albumin, respectively. The associations between liver function tests and the risk of diabetes did not significantly differ by sex in both cohorts (P>0.1 for interaction). In the EPIC-NL case-cohort study, stratified analysis by sex showed that the direction of the association between albumin and diabetes risk was changed in men after adjustment for age, BMI with family history of diabetes (also for the KORA basic model plus glucose) (data not shown).

**Table 2:** Associations of liver function tests with the risk of type 2 diabetes.

	EPIC-NL case-chort study HR (95%) per log2 unit increase			PREVEND cohort study OR (95% CI) per log2 unit increase	
Liver markers	Step 1	Step 2	Step 3	Step 1	Step 2
**Total**					
GGT– U/liter	2.08 (1.94–2.22)	1.49 (1.37–1.62)	1.45 (1.34–1.58)	1.76 (1.60,1.93)	1.22 (1.09–1.38)
ALT– U/liter	2.05 (1.86–2.26)	1.22 (1.09–1.37)	1.05 (0.93–1.18)	1.88 (1.66, 2.14)	1.29 (1.11–1.50)
AST– U/liter	1.81 (1.52–2.16)	0.97 (0.81–1.17)	1.04 (0.87–1.22)	1.63 (1.31, 2.02)	1.16 (0.89–1.50)
Albumin– g/L	0.56 (0.34–0.92)	0.34 (0.21–0.54)	0.42 (0.24–0.74)	0.50 (0.17, 1.49)	0.31 (0.87–1.05)
**Women**					
GGT– U/liter	2.03 (1.89–2.18)	1.45 (1.32–1.58)	1.40 (1.27–1.54)	1.86 (1.60–2.16)	1.22 (1.01–1.49)
ALT– U/liter	1.85 (1.66–2.06)	1.07 (0.94–1.22)	0.92 (0.81–1.05)	1.95 (1.57–2.42)	1.29 (1.00–1.67)
AST– U/liter	1.71 (1.39–2.10)	0.80 (0.64–0.99)	0.85 (0.70–1.04)	1.44 (0.97–2.15)	1.20 (0.76–1.88)
Albumin– g/L	0.49 (0.29–0.82)	0.23 (0.15–0.37)	0.53 (0.31–0.90)	0.45 (0.08–2.42)	0.36 (0.05–2.29)
**Men**					
GGT– U/liter	1.98 (1.68–2.34)	1.43 (1.17–1.76)	1.33 (1.08–1.63)	1.67 (1.48–1.88)	1.22 (1.05–1.42)
ALT– U/liter	2.62 (2.14–3.20)	1.87 (1.48–2.35)	1.64 (1.29–2.08)	1.80 (1.53–2.10)	1.29 (1.07–1.56)
AST– U/liter	2.08 (1.50–2.89)	1.66 (1.16–2.38)	1.73 (1.20–2.51)	1.62 (1.24–2.10)	1.15 (0.84–1.58)
Albumin– g/L	0.44 (0.10–1.97)	4.73 (0.92–24.32)	4.23 (0.83–21.58)	0.38 (0.09–1.63)	0.22 (0.04–1.14)

In step, we adjusted for age and sex (in total populations); step 2, further adjusted for BMI (kg/m2), ex-smoker (yes = 1, no = 0), current smoking (yes = 1, no = 0), parental diabetes (yes = 1, no = 0), hypertension (yes = 1, no = 0), glucose (mmol/l) and uric acid (µmol/l); and model 3 further adjusted for HbA1c (only in the EPIC-NL case-cohort study).

EPIC-NL denotes European Prospective Investigation Into Cancer, PREVEND Prevention of Renal and Vascular End-stage Disease, CI confidence interval.

### Predictive Value of Liver Function Tests

In the EPIC-NL case-cohort study, the basic model showed a C-statistic of 0.823 (0.810–0.837) for the 7.5-year risk of diabetes (Table 2). Addition of liver function tests improved the C-statistic of the basic model (C-statistic change = 0.024; P<0.001) and led to an IDI of 0.011 (P<0.001) and NRI of 9.5% (P<0.001). After addition of each component of liver function tests alone to the basic models, the C-statistic changes were 0.014 (P<0.001), 0.006 (P<0.001), 0.001 (P = 0.15) and 0.002 (P = 0.13) for GGT, ALT, AST and albumin, respectively. Addition of liver function tests also improved prediction for clinical model 1 (C-statistic change = 0.009; P<0.001; NRI = 8.8%; P<0.001). Although addition of liver function tests did not improve prediction for clinical model 2 in the total population (C-statistic change = 0.002; P = 0.61; NRI = 1.2%; P = 0.3), a slight improvement, although not statistically significant in terms of discrimination, was observed when men were considered separately (C-statistic change = 0.007; P = 0.06; NRI = 3.3%; P = 0.04). In women, addition of liver function tests improved prediction for clinical model 1, but did not improve for clinical model 2 (Table 3).

**Table 3:** Incremental predictive value of liver function tests for the risk of type 2 diabetes.

	EPIC-NL case-cohort study				PREVEND cohort study			
Prediction Models	C value (95% CI)	P value	IDI (P value)	NRI (%)(P value)	C value(95% CI)	P value	IDI,P value	NRI (%),P value
**Total sample**								
KORA_Basic_	0.823 (0.810–0.837)	Ref.	Ref.	Ref.	0.775 (0.753–0.793)	Ref.	Ref.	Ref.
KORA_Basic_ + LFTs	0.847 (0.834–0.859)	<0.001	0.011<0.001	9.5, <0.001	0.794 (0.777–0.812)	<0.001	0.017, <0.001	8.6, <0.001
KORA_Basic_ + Glucose + UA_S_	0.894 0.883–0.905)	Ref.	Ref.	Ref.	0.849 (0.833–0.865)	Ref.	Ref.	Ref.
KORA_Basic_ + Glucose + UA_S_ + LFTs	0.903 (0.892–0.913)	<0.001	0.016, <0.001	8.8, <0.001	0.857 (0.841–0.872)	0.003	0.015, <0.001	3.6, 0.03
KORA_Basic_ + Glucose + UA_S_ + HbA1c	0.919 (0.908–0.929)	Ref.	Ref.	Ref.	–	–	–	–
KORA_Basic_ + Glucose + UA_S_ + HbA1c + LFTs	0.922 (0.912–0.932)	0.61	0.002, 0.18	1.2, 0.3	–	–	–	–
**Women**								
KORA_Basic_	0.826 (0.810–0.841)	Ref.	Ref.	Ref.	0.822 (0.795–0.848)	Ref.	Ref.	Ref.
KORA_Basic_ + LFTs	0.848 (0.833–0.862)	<0.001	0.011, <0.001	10.9, <0.001	0.834 (0.808–0.861)	0.01	0.019, <0.001	6.3, 0.03
KORA_Basic_ + Glucose + UA_S_	0.898 (0.886–0.910)	Ref.	Ref.	Ref.	0.883 (0.859–0.907)	Ref.	Ref.	Ref.
KORA_Basic_ + Glucose + UA_S_ + LFTs	0.912 (0.901–0.923)	<0.001	0.013, <0.001	8.1, <0.001	0.886 (0.862–0.910)	0.29	0.013, 0.001	2.2, 0.28
KORA_Basic_ + Glucose + UA_S_ + HbA1c	0.933 (0.923–0.944)	Ref.	Ref.	Ref.	–	–	–	–
KORA_Basic_ + Glucose + UA_S_ + HbA1c + LFTs	0.935 (0.925–0.946)	0.53	0.003, 0.05	1.1, 0.40	–	–	–	–
**Men**								
KORA_Basic_	0.818 (0.788–0.847)	Ref.	Ref.	Ref.	0.724 (0.697–0.750)	Ref.	Ref.	Ref.
KORA_Basic_ + LFTs	0.844 (0.817–0.871)	<0.001	0.10, <0.001	18.3, <0.001	0.755 (0.729–0.781)	<0.001	0.021, <0.001	9.1, 0.003
KORA_Basic_ + Glucose + UA_S_	0.872 (0.847–0.897)	Ref.	Ref.	Ref.	0.809 (0.785–0.832)	Ref.	Ref.	Ref.
KORA_Basic_ + Glucose + UA_S_ + LFTs	0.876 (0.851–0.901)	0.54	0.013, <0.001	6.0, 0.01	0.822 (0.799–0.845)	0.01	0.019, <0.001	5.4, 0.04
KORA_Basic_ + Glucose + UA_S_ + HbA1c	0.877 (0.854–0.905)	Ref.	Ref.	Ref.	–	–	–	–
KORA_Basic_ + Glucose + UA_S_ + HbA1c + LFTs	0.883 (0.860–0.911)	0.06	0.009, 0.01	3.3, 0.04	–	–	–	–

KORA_Basic_ model included data on age, BMI (kg/m2), ex-smoker (yes = 1, no = 0), current smoking (yes = 1, no = 0), parental diabetes (yes = 1, no = 0), hypertension (yes = 1, no = 0).

EPIC-NL denotes European Prospective Investigation Into Cancer, PREVEND Prevention of Renal and Vascular End-stage Disease, CI confidence interval, IDI integrated discrimination improvement, NRI, net reclassification improvement, LFTs, liver function tests (including aspartate aminotransferase, alanine aminotransferase, γ-glutamyl transpeptidase and albumin), KORA Cooperative Health Research in the Region of Augsburg, HbA1c glycated haemoglobin, UA_S_ serum uric acid.

In the PREVEND cohort study, the basic model showed a C-statistic of 0.775 (0.757–0.793). Addition of liver function tests improved the C-statistic of the basic model (C-statistic change = 0.019; P<0.001) and led to an IDI of 0.01 (P<0.001) and NRI of 8.7% (P<0.001). After addition of each component of liver function tests alone to the basic models, the C-statistic changes were 0.013 (P<0.001), 0.011 (P = 0.002), 0.002 (P = 0.29) and 0.0001 (P = 0.98) for GGT, ALT, AST and albumin, respectively. Addition of liver function tests improved prediction for clinical model 1 in the total population (change of C-statistic = 0.008; P = 0.003; NRI = 3.6%; P = 0.03), with the largest change in men (C-statistic change = 0.013; P = 0.01; NRI = 5.4%; P = 0.04) (Table 1). In both cohorts, predictive power slightly increased when we added more liver function tests to the KORA model. For example, in the EPIC-NL study, NRI increased from 6% to 9.5% when we added the panel of all four available liver function tests to the KORA model rather than only the combination of GGT+ALT (Table S2).

In both cohorts, the basic and clinical models provided slightly better discrimination in women than in men. For example, in the EPIC-NL study, the C-statistic of the basic model was 0.826 (0.810–0.841) in women while this was 0.818 (0.788–0.847) in men. For clinical model 2, the C-statistic was 0.933 (0.923–0.944) in women while this was 0.877 (0.854–0.905) in men. In the PREVEND study, the C-statistic of the basic model was 0.822 (0.95–0.848) in women while this was 0.724 (0.697–0.750) in men. For clinical model 1, the C-statistic was 0.883 (0.859–0.907) in women while this was 0.809 (0.785–0.832) in men (Table 3).

In a sensitivity analysis, our results using data only from the MORGEN cohort with less than 10% missing values for non-fasting glucose were comparable with our results using both cohorts of the EPIC-NL study. Addition of liver function tests improved the C-statistic of the basic KORA model (C-statistic change = 0.020; P<0.001) and led to an IDI of 0.006 (P<0.001) and NRI of 9.3% (P<0.001). Addition of liver function tests did not improve prediction for clinical model 2 (C-statistic change = 0.004, P = 0.10; IDI = 0.003, P = 0.11; NRI = 2.2%, P = 0.20).

## Discussion

In this prospective analysis, we examined whether addition of liver function tests could be useful to improve prediction of developing type 2 diabetes above the basic and clinical models in two independent large population-based cohorts. We observed that addition of liver function tests improved prediction modestly only for a basic model without biomarkers in terms of discrimination and reclassification in each cohort. Furthermore, addition of liver function tests led to small but statistically significant improvements in prediction based on a clinical model incorporating glucose and serum uric acid, but not if the clinical model also includes HbA1c. However, there was a slightly better improvement in prediction for men.

Several studies have been performed to investigate the associations of liver function tests with type 2 diabetes and its related outcomes [1], [4]. However, just a limited number of studies aimed to examine the incremental predictive value of these markers over available prediction models. A analysis of the EPIC-Potsdam cohort showed that a combination of triglycerides, HDL- cholesterol, GGT and ALT further improved prediction based on the German diabetes risk score incorporating glucose and HbA1c in the same population in which they had previously developed that risk score [5]. In the DESIR study, a model with GGT and glucose showed an improved prediction in men compared with a basic model incorporating data on smoking, waist circumference and hypertension [6]. Recently, the British Heart Study showed that a clinical model incorporating GGT plus HbA1c improved prediction compared with a basic simple model. However, addition of GGT itself had little improvement above a clinical model incorporating glucose, HDL cholesterol and triglyceride [7]. Of note, it is particularly important for the value of biomarkers to be examined in an independent setting, because the improvement in measures of prediction can be overestimated if the same population is used for development and evaluation of the incremental value of new biomarkers [10], [28], [29]. In our study, we scientifically evaluated incremental predictive value of liver function tests in two independent Dutch populations because we intended to validate our findings in another setting as well. In this way, we took advantage of using a different case mix and slightly different measurement of diabetes between two cohorts [28].

Furthermore, we also did this analysis for women and men separately to take into account potential sex differences in the risk prediction of diabetes [6], [30]. For example, we and others have shown that prediction models might have a slightly better performance to identify women at high risk [6], [11], [13]. We observed no differences in the incremental predictive value of liver function tests above the basic and clinical model incorporating glucose plus uric acid between women and men. However, there was a statistically significant improvement in prediction only in men when we added liver function tests to a clinical model incorporating glucose plus uric acid plus HbA1c. At population level, it is true that a prediction model, like the KORA basic model, incorporting 6 predictors, performs well to identify the individuals at high risk of future diabetes for 7.5 years. In our study, addition of liver function tests did hardly result in any improvement of prediction once additional data on glycaemia indices were included. The reason why improvements in predictions were limited in the latter clinical models is that the glycaemia indices are integral parts of the clinical outcome of interest, i.e., diabetes. Diabetes itself is defined by certain cut-offs for glucose and/or HbA1c [31].

Like previous studies [1], [4], we demonstrated significant associations of some components of liver function tests with the risk of type 2 diabetes. The associations were independent of common risk factors but addition of liver function tests only minimally to modestly improve the risk prediction of disease. In other words, the absolute difference of certain (bio)markers between individuals who develop and those who remain free of diabetes at a population level is not likely to resolve whether a (bio)marker can be useful for prediction [29]. In fact, on an individual level, the range of marker levels between cases and non-cases overlap, limiting its incremental predictive value [29], [32]. In contrast, although a certain (bio)marker does not show statistical significance in an etiologic relation, it might still have incremental predictive value in combination with other predictors. So from that point of view it is reasonable to examine all four components of liver function tests in each model.

All the basic and clinical models showed slightly better discrimination performance in the EPIC-NL case-cohort study than in the PREVEND cohort study overall and particularly in men. This difference might be explained by differences in heterogeneity between these two populations [33]. Larger heterogeneity between individuals make it easier to differentiate between those at high and low risk and may thus lead to higher C-statistics. For example, variables like age and sex may have larger heterogeneity in the EPIC-NL cohort when compared with the PREVEND cohort.

Another explanation for this is that we ascertained incident cases differently in each cohort. Therefore, we adjusted the KORA basic model for this difference in incidence of diabetes between development population and both of our populations. Although C-statistics are insensitive to error in average outcome, different ascertainment of outcome might have affected discrimination performance of models [22]. However, the incremental predictive value of liver function tests was comparable above each model for both cohorts. It is worthy to mention that our findings are in line with prior evidence on this topic showing minimal to modest prediction improvement for risk of future diabetes [5], [7]. As a general limitation, we should mention that the reclassification improvement is strongly determined by the cut-off values for the risk categories. As we have previously explained [34], in the diabetes prediction the clinically-relevant cut-off values are not clearly stated yet. In fact, it is hard to judge the clinical utility of liver function test at this time. Diagnosis of diabetes is always challenging in observational studies because indivduals with type 2 diabetes may remain undiagnosed for several months to years. Since we used data of self-reports, some cases of type 2 diabetes may have been undetected. Finally, the PREVEND cohort was enriched with individuals with a higher urinary albumin concentration. Therefore, we performed weighted analysis to be able to generalize our findings to the general population. Further studies are warranted to replicate current findings for long-term risk and subsequently evaluate the incremental value of liver function tests [10], [28].

We conclude that a combination of liver function tests can modestly improve prediction of medium-term risk of type 2 diabetes above the basic risk model and the clinical model incorporating data on glucose and serum uric acid. If data on HbA1c are available, these markers of liver injury are of little added predictive value. A slightly better improvement in prediction may be present in men.

## Supporting Information

Figure S1 Calibration plots for comparison of the predicted 7.5-year risk of diabetes (according to the KORA basic model) against observed risk of developing type 2 diabetes. Panel A (the EPIC-NL case-cohort study), Panel B (the PREVEND cohort study). The ‘ideal’ and ‘non-parametric’ terms, the dashed line denotes the ideal calibration line (slope = 1, intercept = 0) and the dotted line denotes smooth calibration curve for each models. Hosmer-Lemeshow χ2 statistic were 14.7 (P = 0.10) and 7.8 (P = 0.56) for the calibration performance of KORA basic model (after adjustment for the intercept and the slope) in the EPIC-NL and in the PREVEND studies, respectively.
(DOC)

Table S1 Missing data pattern in extrapolated EPIC-NL case-cohort study and PREVEND cohort study.
(DOC)

Table S2 Incremental predictive value of components of liver function tests for the risk of future type 2 diabetes.
(DOC)
