# Supplementary material for: Liver Function Tests and Risk Prediction of Incident Type 2 Diabetes: Evaluation in Two Independent Cohorts
Source: PLoS One. 2012 Dec 17;7(12):e51496. doi: 10.1371/journal.pone.0051496 (PMC3524238; doi:10.1371/journal.pone.0051496)
Supplement: Figure S1 — Calibration plots for comparison of the predicted 7.5-year risk of diabetes (according to the KORA basic model) against observed risk of developing type 2 diabetes. Panel A (the EPIC-NL case-cohort study), Panel B (the PREVEND cohort study). The ‘ideal’ and ‘non-parametric’ terms, the dashed line denotes the ideal calibration line (slope = 1, intercept = 0) and the dotted line denotes smooth calibration curve for each models. Hosmer-Lemeshow χ2 statistic were 14.7 (P = 0.10) and 7.8 (P = 0.56) for the calibration performance of KORA basic model (after adjustment for the intercept and the slope) in the EPIC-NL and in the PREVEND studies, respectively. (DOC) [file pone.0051496.s001.doc]

**Figure S1. Calibration plots for comparison of the predicted 7.5-year risk of diabetes (according to the KORA basic model) against observed risk of developing type 2 diabetes.**

Panel A (the EPIC-NL case-cohort study), Panel B (the PREVEND cohort study). The ‘ideal’ and ‘non-parametric’ terms, the dashed line denotes the ideal calibration line (slope=1, intercept=0) and the dotted line denotes smooth calibration curve for each models. Hosmer-Lemeshow χ^2^ statistic were 14.7 (P=0.10) and 7.8 (P=0.56) for the calibration performance of KORA basic model (after adjustment for the intercept and the slope) in the EPIC-NL and in the PREVEND studies, respectively.

**A**

**B**
