# Supplementary material for: Liver Function Tests and Risk Prediction of Incident Type 2 Diabetes: Evaluation in Two Independent Cohorts
Source: PLoS One. 2012 Dec 17;7(12):e51496. doi: 10.1371/journal.pone.0051496 (PMC3524238; doi:10.1371/journal.pone.0051496)
Supplement: Table S1 — Missing data pattern in extrapolated EPIC-NL case-cohort study and PREVEND cohort study. (DOC) [file pone.0051496.s002.doc]

1. **Table S1. Missing data pattern in extrapolated EPIC-NL case-cohort study and PREVEND cohort study**

|  | **Extrapolated**  **EPIC-NL case-cohort study** | | **PREVEND cohort study** | |
| --- | --- | --- | --- | --- |
| **Variables** | Missing values | Percent | Missing values | Percent |
| Age | 0 | 0 | 0 | 0 |
| Sex | 0 | 0 | 0 | 0 |
| Incident type 2 diabetes | 0 | 0 | 0 | 0 |
| Weight | 20 | 0.05 | 77 | 1.0 |
| Height | 24 | 0.06 | 77 | 1.0 |
| Body Mass Index | 25 | 0.06 | 77 | 1.0 |
| Smoking | 153 | 0.4 | 22 | 0.3 |
| Family history of diabetes | 2234 | 5.8 | 601 | 7.5 |
| Systolic blood pressure | 90 | 0.2 | 2 | 0.02 |
| Diastolic blood pressure | 66 | 0.17 | 2 | 0.02 |
| History of hypertension | 2462 | 6.4 | 160 | 2.0 |
| Antihypertensive medication | 0 | 0 | 20 | 0.3 |
| Glucose | 7889 | 20.5 | 54 | 0.7 |
| HbA1c | 2748 | 7.2 | - | - |
| Uric acid | 2542 | 6.6 | 195 | 2.4 |
| GGT | 2565 | 6.7 | 1110 | 13.9 |
| AST | 2565 | 6.7 | 1080 | 13.5 |
| ALT | 2565 | 6.7 | 1080 | 13.5 |
| Albumin | 2565 | 6.7 | 1081 | 13.5 |

EPIC-NL denotes European Prospective Investigation Into Cancer (the Netherlands), PREVEND Prevention of Renal and Vascular End-stage Disease, AST, aspartate aminotransferase, ALT, alanine aminotransferase, GGT, γ-glutamyl transpeptidasein, HbA1c glycated haemoglobin.
