# Supplementary material for: Liver Function Tests and Risk Prediction of Incident Type 2 Diabetes: Evaluation in Two Independent Cohorts
Source: PLoS One. 2012 Dec 17;7(12):e51496. doi: 10.1371/journal.pone.0051496 (PMC3524238; doi:10.1371/journal.pone.0051496)
Supplement: Table S2 — Incremental predictive value of components of liver function tests for the risk of future type 2 diabetes. (DOC) [file pone.0051496.s003.doc]

**Table S2.** Incremental predictive value of components of liver function tests for the risk of future type 2 diabetes

|  | **EPIC-NL case-cohort study** | | | | **PREVEND cohort study** | | | |
| --- | --- | --- | --- | --- | --- | --- | --- | --- |
| **Prediction Models** | **C value**  **(95% CI)** | **P value** | **IDI**  **(P value)** | **NRI (%)**  **(P value)** | **C value**  **(95% CI)** | **P value** | **IDI,**  **P value** | **NRI (%),**  **P value** |
| **Total sample** |  |  |  |  |  |  |  |  |
| KORA_Basic_ | 0.823  (0.810-0.837) | Ref. | Ref. | Ref. | 0.775  (0.753-0.793) | Ref. | Ref. | Ref. |
| KORA_Basic_ + GGT | 0.837  (0.824-0.849) | <0.001 | 0.003  0.002 | 5.8,  <0.001 | 0.789  (0.772-0.807) | <0.001 | 0.010,  <0.001 | 5.0,  0.006 |
| KORA_Basic_ + ALT | 0.829  0.816-0.841) | 0.001 | -0.0004  0.82 | 1.5  0.24 | 0.786  (0.768-0.804) | 0.002 | 0.010,  <0.001 | 4.0  0.03 |
| KORA_Basic_  + AST | 0.822  (0.809-0.835) | 0.13 | 0.004,  0.001 | -0.4  0.47 | 0. 776  (0.758-0.794) | 0.58 | 0.003,  0.03 | 2.0,  0.16 |
| KORA_Basic_ + ALB | 0.825  (0.812-0.839) | 0.07 | 0.003  0.001 | 1.3  0.28 | 0.775  (0.757-0.793) | 0.94 | 0.0004  0.52 | -0.6  0.26 |
| KORA_Basic_  + ALT+ GGT | 0.837  (0.824-0.850) | <0.001 | 0.002,  0.004 | 6.0,  <0.001 | 0.790  (0.772-0.808) | <0.001 | 0.012  <0.001 | 4.8  0.02 |
| KORA_Basic_  + LFTs | 0.847  (0.834-0.859) | <0.001 | 0.011  <0.001 | 9.5,  <0.001 | 0.794  (0.777-0.812) | <0.001 | 0.017,  <0.001 | 8.6,  <0.001 |

KORA_Basic_ model included data on age, BMI (kg ⁄m^2^), ex-smoker (yes = 1, no = 0), current smoking (yes = 1, no = 0), parental diabetes (yes = 1, no = 0), hypertension (yes = 1, no = 0).

EPIC-NL denotes European Prospective Investigation Into Cancer, PREVEND Prevention of Renal and Vascular End-stage Disease, CI confidence interval, IDI integrated discrimination improvement, NRI, net reclassification improvement, LFTs, liver function tests (including aspartate aminotransferase, alanine aminotransferase, γ-glutamyl transpeptidase and albumin), KORA Cooperative Health Research in the Region of Augsburg, HbA1c glycated haemoglobin, UA_S_ serum uric acid.
